# *Coelogyne putaoensis* (Orchidaceae), a new species from Myanmar

**DOI:** 10.3897/phytokeys.82.13172

**Published:** 2017-06-29

**Authors:** Ye Lwin Aung, Xiaohua Jin, André Schuiteman

**Affiliations:** 1 State Key Laboratory of Systematic and Evolutionary Botany, Institute of Botany, Chinese Academy of Sciences, Beijing 100093, China; 2 Southeast Asia Biodiversity Research Institute, Chinese Academy of Sciences, Yezin, Nay Pyi Taw 05282, Myanmar; 3 Science Directorate, Royal Botanic Gardens, Kew, Richmond, Surrey TW9 3AB, U.K.

**Keywords:** Arethuseae, Kachin, key, montane forest, section *Ocellatae*, taxonomy

## Abstract

*Coelogyne putaoensis*, a new species of section Ocellatae from Putao, Kachin State, Myanmar, is described and illustrated. It is morphologically similar to *C. taronensis* and *C. weixiensis*, presumably its nearest relatives. An identification key and colour photographs are provided. A preliminary risk-of-extinction assessment according to the IUCN Red List categories and criteria is given for the new species.

## Introduction

*Coelogyne* Lindl. (Lindley 1821) (Orchidaceae, Epidendroideae, Arethuseae) is a genus of about 200 species, distributed from South and Southeast Asia into the Pacific as far east as Fiji and Samoa (Clayton 2002, Chen et al. 2009, George and George 2011, Gravendeel 2005). Around 45 species of *Coelogyne* have been recorded from Myanmar (Kress et al. 2003; Kurzweil and Lwin 2014). During fieldwork in Putao, Kachin State, Northern Myanmar, in June 2016, the first authors discovered a new species of *Coelogyne*, which is described below. The new species belongs to Coelogyne section Ocellatae Pfitzer & Kraenzl. (Pfitzer and Kraenzl. 1907).

## Material and methods

All measurements of the three species here discussed, i.e., *Coelogyne putaoensis*, *C. taronensis* Handel-Mazzetti and *C. weixiensis* X.H. Jin, were taken from dried herbarium specimens and field notes. In the description, length and width are represented as length × width. About twenty living plants and three dried specimens of the new species and 10 specimens each of *C. taronensis* and *C. weixiensis*, including types or photos of types of all taxa, were examined.

## Taxonomic treatment

### Coelogyne putaoensis

Taxon classificationPlantaeAsparagalesOrchidaceae

X.H. Jin, L.A. Ye & Schuit. sp. nov.

urn:lsid:ipni.org:names:77163812-1

Figures 1 , 2 , 3

#### Diagnosis.

*Coelogyne putaoensis* is similar to *C. taronensis* and *C. weixiensis*, but can be distinguished by its solid yellowish brown sepals and petals, a brown lip with bright yellow markings, three keels extending from the base of the lip onto the mid-lobe, and lateral keels adorned with papillae.

**Figure 1.**
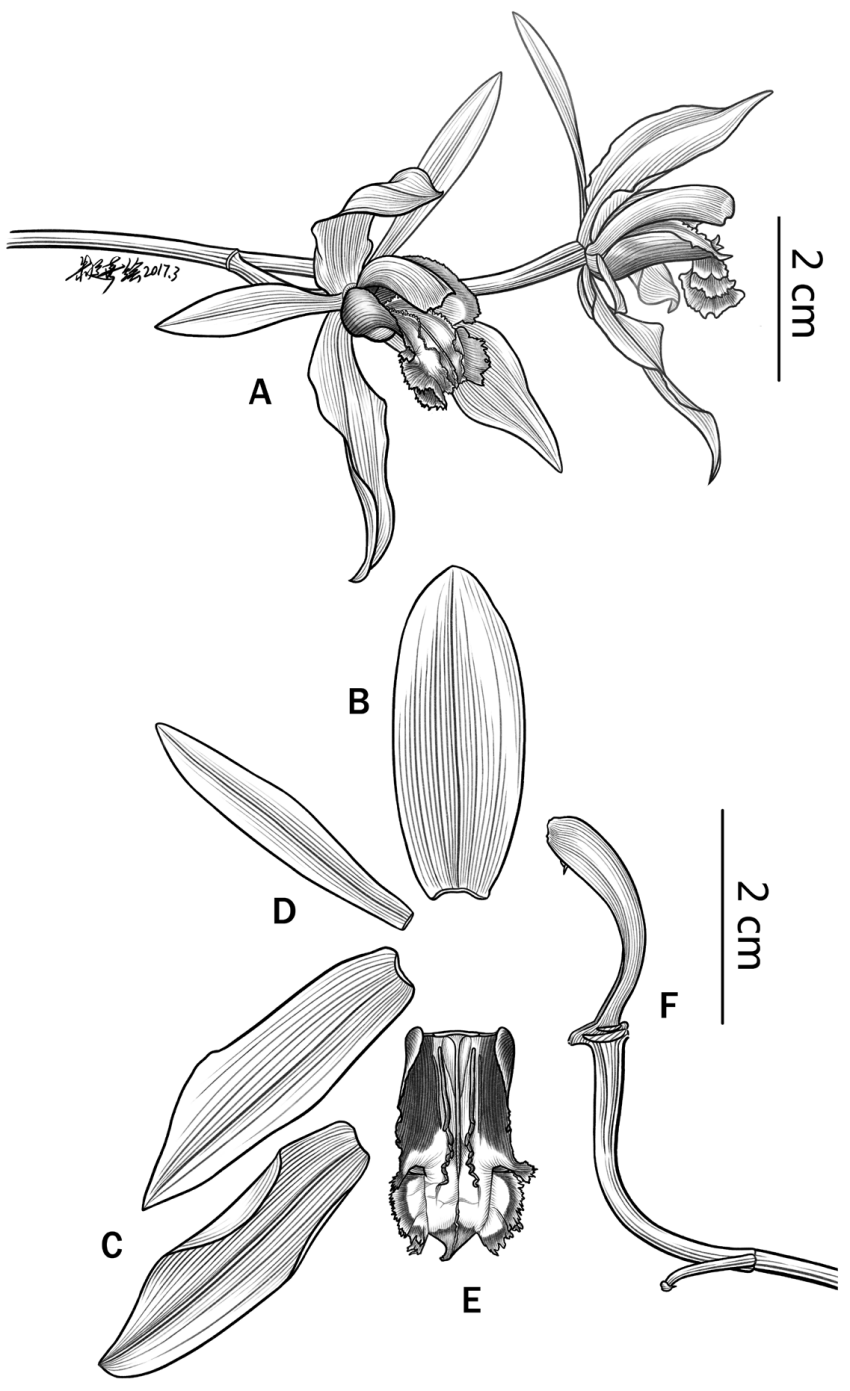
*Coelogyne putaoensis* X.H. Jin, L.A. Ye & Schuit. **A** Inflorescence **B** Dorsal sepal **C** Lateral sepals **D** Petal **E** Lip **F** Lateral view of column. Illustration by Yunxi Zhu.

#### Type.

MYANMAR. Kachin State: Putao Township, Hponkanrazi Wildlife Sanctuary, subtropical, evergreen, broad-leaved, montane forest, 2500–3100 m, epiphytic on tree trunks or lithophytic on rocks, 14 June 2016, *Xiaohua Jin et al, PT-2116* (Holotype, PE!).

**Figure 2.**
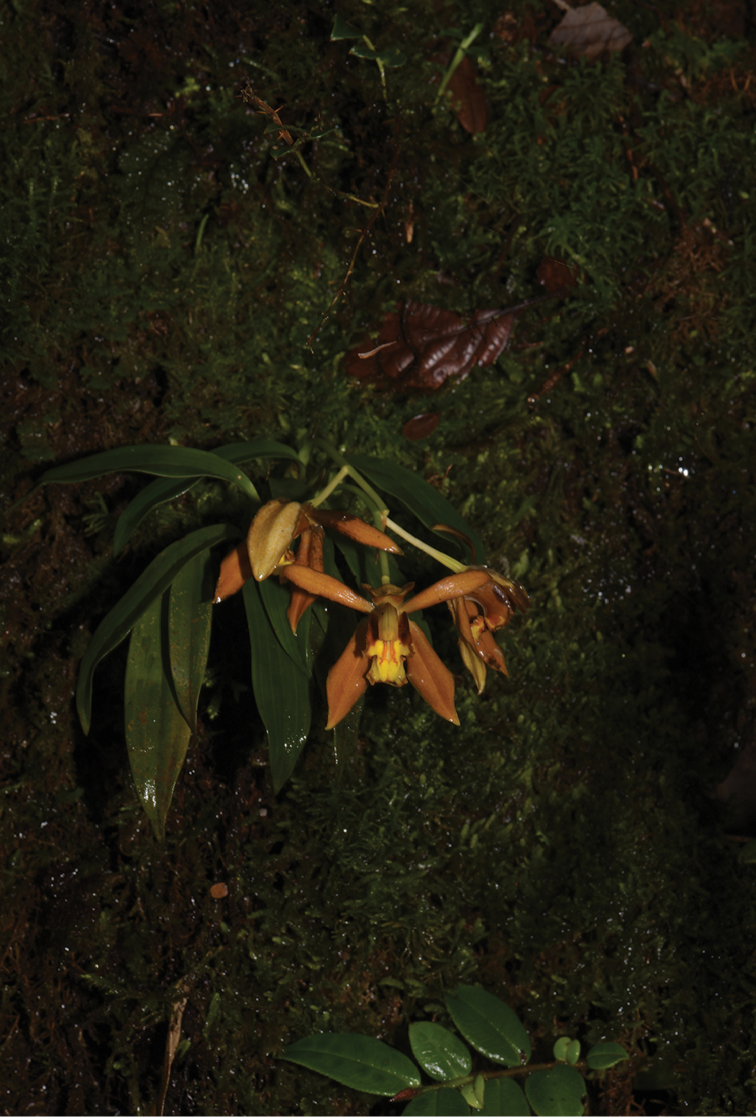
Habit of *Coelogyne putaoensis*. Photo by X.H. Jin.

#### Description.

Pseudobulbs crowded on a short and stout rhizome, ovoid, 2.5–3.2 × 0.8–0.9 cm, when mature covered with brown sheaths at the base, bifoliate. Leaves erect, petiolate, ovate-lanceolate, 6.5–9.5 × 1.2–1.8 cm, including the ca. 1 cm long petiole, acute to acuminate, coriaceous, with 5–7 veins. Inflorescence proteranthous, peduncle arching, 2–3.5 cm long, rachis slender, 2.5 cm long, 2- to 3- flowered. Flowers yellowish brown, up to 6 cm across, lip adaxially on the mid-lobe with a large, bright yellow patch, connected to a bright yellow blotch on the front part of each of the side lobes, the keels orange-brown with much paler, almost whitish marginal papillae. Pedicel-with-ovary 2.2–2.5 cm long, glabrous. Dorsal sepal narrowly elliptic, 3.2 × 1.3 cm, 7-veined, acute. Lateral sepals oblique, oblong-lanceolate, 3.5 × 1 cm, 7-veined, acuminate. Petals narrowly oblanceolate, 2.9 × 0.5 cm, 5-veined, acute at apex, clawed at base. Lip 3-lobed, 2.7 × 1.6 cm; lateral lobes rounded, erect; mid-lobe triangular, 1.6 × 1 cm, margin undulate and lacerate; callus of three keels, extending from the base of lip to the middle (lateral keels) or apex of the mid-lobe (central keel), the central one lower than the lateral two on the mid-lobe, margins of the lateral keels adorned with papillae. Column arching, winged at apex, 1.9 cm long. Fruit not seen.

**Figure 3.**
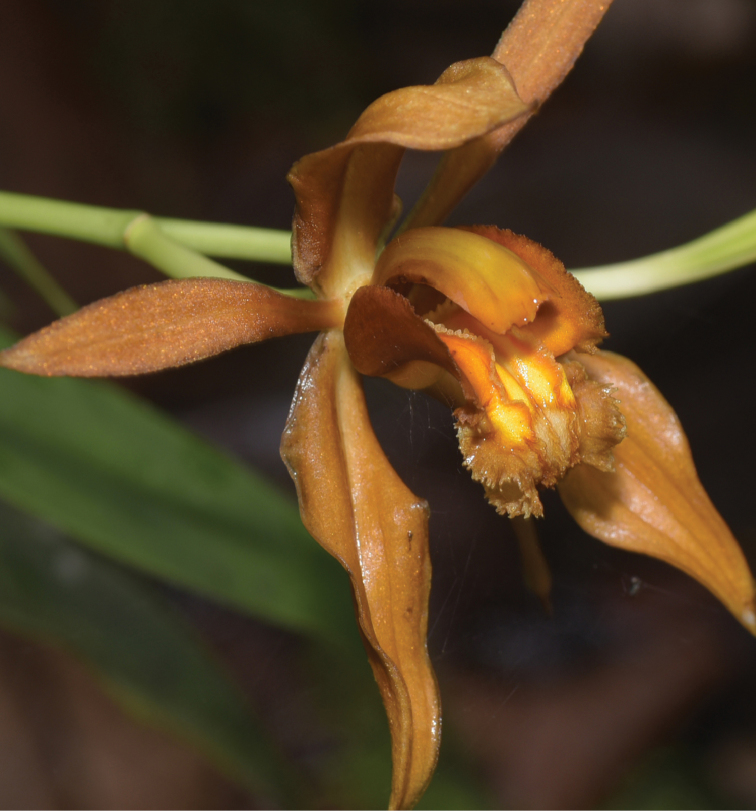
Close-up of flower of *Coelogyne putaoensis*, showing the white papillae on the two lateral lamellae. Photo by X.H. Jin.

#### Etymology.

The new species is named after Putao, the northernmost town of Myanmar near which it was discovered in a vast area of unspoiled mountain forest.

#### Distribution and habitat.

*Coelogyne putaoensis* is a predominantly epiphytic species that grows on moss-covered branches and tree trunks, sometimes also on rocks, in humid, broad-leaved, evergreen, montane forest, from 2500 to 3100 m elevation. At present, *C. putaoensis* is only known from the type locality.

#### Conservation status.

**Least Concern (LC).** *Coelogyne putaoensis* was collected in the Hponkanrazi Wildlife Sanctuary, Putao, Northern Myanmar. Until now, only one population, consisting of ca. 200 individuals, has been discovered in the reserve (1044 square miles), which is a legally protected area under the management of the Myanmar Forest Department. As no threat currently affects the quality of its habitat and the number of mature individuals, the species is here assigned a preliminary status of Least Concern (LC) according to the guidelines for using the IUCN Red List Categories and Criteria (IUCN Standards and Petitions Subcommittee 2017).

#### Key to *Coelogyne putaoensis*, *C. taronensis* and *C. weixiensis*

**Table:** 

1	Flowers almost solid yellowish brown, except for yellow patches on the lip; lip with three lamellate keels, all extending onto the mid-lobe, the lateral ones with marginal papillae	***Coelogyne putaoensis***
–	Flowers white, light greenish or light yellow, sepals and petals with or without darker veins, lip with two or four orange to red-brown blotches (resembling eye-spots) and with two or three lamellate keels, the central one, if present, not extending onto the mid-lobe; the lateral ones without marginal papillae	**2**
2	Sepals and petals white or greenish white, without coloured veins; dorsal sepal 11-veined; lip usually with 4 eye-like yellow blotches bordered with brown, two on the mid-lobe and two on the side-lobes; mid-vein white	***Coelogyne taronensis***
–	Sepals and petals yellowish with orange-brown veins; dorsal sepal 7–9-veined; lip with two solid brown spots on the side-lobes, sometimes with an orange patch between the keels on the mid-lobe; mid-vein brown	***Coelogyne weixiensis***

## Discussion

**Figure 4.**
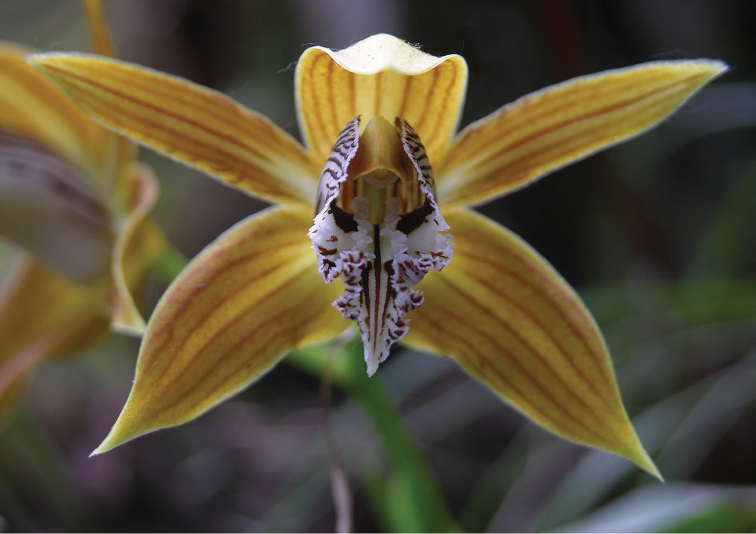
Close-up of flower of *Coelogyne weixiensis*. Photo by X.H. Jin.

**Figure 5.**
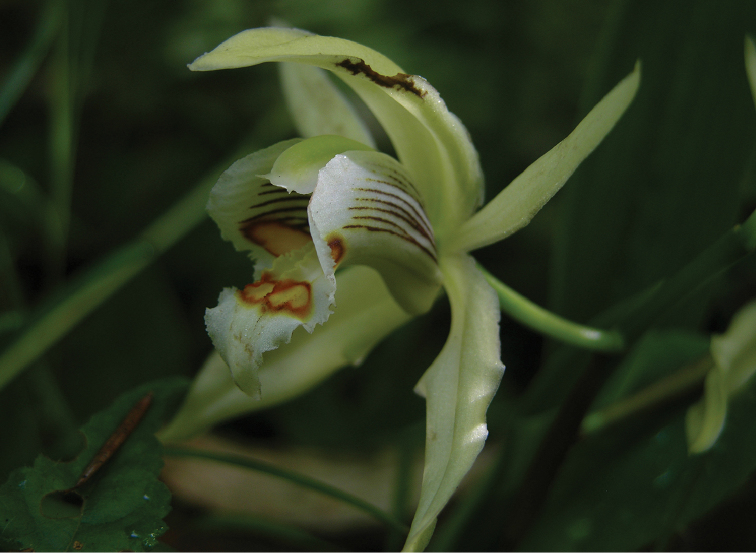
Close-up of flower of *Coelogyne taronensis*. Photo by X.H. Jin.

*Coelogyne taronensis*, *C. weixiensis* and *C. putaoensis* are similar, both in vegetative morphology and in the size and shape of the flowers, and all come from the same general region, northern Myanmar and adjacent parts of China. There can be little doubt that they are closely related. They are readily distinguished on the basis of their colour differences, as indicated in the key and photos. *Coelogyne weixiensis* (Figure 4) and *C. taronensis* (Figure 5) are especially similar morphologically. They are listed as synonyms by George and George (2011). At present, too little is known about the variability of *C. taronensis* to make any assessment with confidence. Both taxa appear to be rare and local (*C. weixiensis*: China, Yunnan, Bilou Snow Mountains near Weixi; *C. taronensis*: China, Yunnan, Taron (=Dulong) Valley). *Coelogyne putaoensis* differs from the two others not only in having almost solid brown flowers, but also in the papillose margins of the keels on the lip, and in the median keel extending onto the mid-lobe. Altitudinal range and habitat are comparable for the three species: *C. putaoensis* was collected at 2500–3100 m, *C. taronensis* at 2400–3500 m, and *C. weixiensis* at 2600–3000 m elevation (Jin 2005). All three occur as epiphytes in montane forest. At least two of the three species (*C. taronensis* and *C. weixiensis*) are of high conservation interest, and habitat protection seems urgently needed for these. As far as we can ascertain, only *C. weixiensis* is currently in cultivation, having recently received a Botanical Certificate of the Royal Horticultural Society in the UK (as *C. taronensis*).

## Supplementary Material

XML Treatment for Coelogyne putaoensis
